# Modulation of Food Intake by Differential TAS2R Stimulation in Rat

**DOI:** 10.3390/nu12123784

**Published:** 2020-12-10

**Authors:** Carme Grau-Bové, Alba Miguéns-Gómez, Carlos González-Quilen, José-Antonio Fernández-López, Xavier Remesar, Cristina Torres-Fuentes, Javier Ávila-Román, Esther Rodríguez-Gallego, Raúl Beltrán-Debón, M Teresa Blay, Ximena Terra, Anna Ardévol, Montserrat Pinent

**Affiliations:** 1 MoBioFood Research Group, Department of Biochemistry and Biotechnology, Universitat Rovira i Virgili, 43007 Tarragona, Spain; carme.grau@urv.cat (C.G.-B.); alba.miguens@urv.cat (A.M.-G.); carlosalberto.gonzalez@urv.cat (C.G.-Q.); esther.rodriguez@urv.cat (E.R.-G.); raul.beltran@urv.cat (R.B.-D.); mteresa.blay@urv.cat (M.T.B.); ximena.terra@urv.cat (X.T.); montserrat.pinent@urv.cat (M.P.); 2 Department of Biochemistry and Molecular Biomedicine, Faculty of Biology, University of Barcelona, Av. Diagonal 643, 08028 Barcelona, Spain; josfernandez@ub.edu (J.-A.F.-L.); xremesar@ub.edu (X.R.); 3 CIBER Obesity and Nutrition, Institute of Health Carlos III, Av. Diagonal 643, 08028 Barcelona, Spain; 4 Nutrigenomics Research Group, Department of Biochemistry and Biotechnology, Universitat Rovira i Virgili, 43007 Tarragona, Spain; cristina.torres@urv.cat (C.T.-F.); franciscojavier.avila@urv.cat (J.Á.-R.)

**Keywords:** TAS2R5, TAS2R39, TAS2R14, agonist, food intake, GLP1, CCK, PYY

## Abstract

Metabolic surgery modulates the enterohormone profile, which leads, among other effects, to changes in food intake. Bitter taste receptors (TAS2Rs) have been identified in the gastrointestinal tract and specific stimulation of these has been linked to the control of ghrelin secretion. We hypothesize that optimal stimulation of TAS2Rs could help to modulate enteroendocrine secretions and thus regulate food intake. To determine this, we have assayed the response to specific agonists for hTAS2R5, hTAS2R14 and hTAS2R39 on enteroendocrine secretions from intestinal segments and food intake in rats. We found that hTAS2R5 agonists stimulate glucagon-like peptide 1 (GLP-1) and cholecystokinin (CCK), and reduce food intake. hTAS2R14 agonists induce GLP1, while hTASR39 agonists tend to increase peptide YY (PYY) but fail to reduce food intake. The effect of simultaneously activating several receptors is heterogeneous depending on the relative affinity of the agonists for each receptor. Although detailed mechanisms are not clear, bitter compounds can stimulate differentially enteroendocrine secretions that modulate food intake in rats.

## 1. Introduction

Controlling food intake is a complex process that requires the combination of signals with very different origins. In animals, the nervous and hormonal systems play a role but in humans, feelings and sensations due to other environmental factors are also involved [1]. To study and monitor the regulation of food intake, numerous approaches involving diet, physical activity, medical devices, pharmacotherapy and metabolic (bariatric) surgery have been applied.

One of the most effective treatments against obesity and associated metabolic disorders is metabolic surgery [2], which leads to a huge change in metabolism and modifies the gastrointestinal secretome of patients. Enterohormones reach several targets in the body, including the brain and other peripheral tissues (e.g., adipose tissue, muscle and the gastrointestinal tract) [3]. The most consolidated effect of bariatric surgery in gastrointestinal secretome is an increase in glucagon-like peptide 1 (GLP-1) and peptide YY (PYY) [4]. Reproducing this enterohormone modulation without surgery could produce some of the beneficial effects of surgery without being so invasive. Several nutritional approaches cause enterohormone profile regulation [5]. However, they are not always effective at controlling food intake, probably because there is little control over the composition of the food components.

The bitter taste helps us to protect ourselves against unhealthy natural products [6]. Nevertheless, not all toxins are bitter and not all bitter compounds are toxic [7]. In fact, many bitter compounds have health benefits. It has even been suggested that healthier diets contain a higher proportion of bitter-tasting ingredients, such as bitter vegetables [8]. Recently, bitter taste receptors (TAS2Rs) have been identified in locations other than the mouth—where taste perception occurs—but a clear role for them there has not yet been defined [9,10]. Meyerhof et al. studied the association between bitter ligands and specific TAS2Rs using in vitro assays and calcium imaging [11]. Together with Di Pizio [12], they showed that humans and mice [13], which have a relatively large number of bitter taste receptors (25 in humans and 35 in mice), contain different types of receptors depending on their selectivity. Some selective receptors (such as hTAS2R3) only bind 1–3 ligands. In contrast, less selective receptors (such as hTAS2R39) and highly promiscuous receptors bind several ligands. A promiscuous TAS2R is one that can be activated by several ligands. In turn, ligands can be specific or unspecific for a certain receptor [12]. Species with a more limited number of bitter taste receptors contain only promiscuous receptors. Research is currently being conducted into the role of these bitter taste receptors that are located away from the lingual papillae where taste is perceived. They have been found in several locations, including the lungs [14] or stomach. Our group and others have shown that stimulating them induces ghrelin secretion in the murine ghrelinoma cell line [15] and in human fundic cells [16].

In this paper, we test whether a profile of enterohormones that limits food intake can be obtained by stimulating bitter taste receptors differentially. To do so, we used specific ligands for bitter taste receptors with different ranges of specificity, according to Meyerhof’s definitions [11,17]. We also tested the effects of these ligands on enterohormone secretions in various segments of the rat intestine. Finally, we associated their effects on rat gastrointestinal secretome with their ability to modulate food intake in the whole animal.

## 2. Materials and Methods

### 2.1. Chemicals and Reagents

1,10-Phenantroline, (-)-epicatechin, Thiamine, Flufenamic acid, Vanillic acid and Protocatechuic acid were purchased from Sigma (Barcelona, Spain). Procyanidin B2 gallate, Epigallocatechin gallate (EGCG) and epicatechin gallate were purchased from Extrasynthese (Genay, France) and Procyanidin B2 was purchased from Adooq-Bioscience (Irvine, CA, USA).

We used Krebs–Ringer bicarbonate (KRB) buffer (Hepes 11.5 mM, CaCl_2_ 2.6 mM, MgCl_2_ 1.2 mM, KCl 5.5 mM, NaCl 138 mM, NaHCO_3_ 4.2 mM, NaH_2_PO_4_ 1.2 mM) pH 7.4, supplemented with either 10 mM D-Glucose (KRB-D-Glucose buffer) or 10 mM D-Mannitol (KRB-D-Mannitol buffer). For enterohormone secretion studies, KRB-D-Glucose was supplemented with protease inhibitors: 10 µM amastatin (Enzo Life Sciences, Madrid, Spain), 100 KIU aprotinin (Sigma, Barcelona, Spain) and 0.1% fatty acid free-bovine serum albumin.

### 2.2. Animals

We used 26 male Fischer-344 rats (Charles River Laboratories, Barcelona, Spain) and 20 female Wistar rats (Envigo, Barcelona, Spain). Most of these animals were housed at the animal housing facility of the Universitat Rovira i Virgili. Ten female Wistar rats were bred and housed at the Faculty of Biology of the Universitat de Barcelona. All rats were housed under standard conditions, i.e., they were caged in pairs at a room temperature of 23 °C with a standard 12-h light-dark cycle (lights on at 7 am), with ventilation, ad libitum access to tap water and a standard chow diet. The Fischer-344 rats were fed with a standard chow diet by SAFE (Cat No: A04, SAFE, Augy, France) while the Wistar rats were fed with a standard chow diet by Teklad (Cat No: Teklad 2014, Envigo, Barcelona, Spain). All procedures were approved by the Experimental Animal Ethics Committee of the autonomous government of Catalonia, Spain (Ministry of Territory and Sustainability, Directorate-General for Environmental Policy and the Natural Environment, project authorization code 10715) and the University of Barcelona (Ministry of Territory and Sustainability, Directorate-General for Environmental Policy and the Natural Environment, project authorization code 10769).

### 2.3. Ex Vivo Treatment of Intestinal Segments

We used 26 male Fischer-344 rats weighing 350–400 g. After a short fasting period (1–3 h), the rats were euthanized by decapitation and their intestines were excised. Samples were collected from the proximal duodenum and distal ileum. The tissue was rinsed with ice-cold KRB-D-Mannitol buffer and dissected into segments (0.5 cm diameter). After 15 min of washing, the tissue segments were placed in prewarmed (37 °C) KRB-D-Glucose buffer 0.1% Dimethyl Sulfoxide (DMSO) containing the compounds to be tested. Duodenal and ileal segments were treated with different compounds or a mix of compounds (Table S1) in a humidified incubator at 37 °C, 95% O_2_ and 5% CO_2_. After 30 min of treatment, the whole volume was frozen and stored at −80 °C for enterohormone quantification.

### 2.4. Studies of Food Intake

Ten female Wistar rats were housed in pairs for one week of adaptation. After this adaptation period, the animals were housed in single cages, introduced to daily 4 h food deprivation before light offset (3 p.m. to 7 p.m.) to habituate them to the experimental schedule, and trained for intragastric oral gavaging with tap water 1 h before dark onset (6 p.m.). One experiment per week was performed in a cross-over design for all food intake studies. For each experiment, the trained animals were treated with different compounds or a mix of compounds at defined concentrations (see Supplementary Table S1) intragastrically by oral gavage 1 h before dark onset (6 p.m.) using tap water as a vehicle. Parallel controls were performed by administering the vehicle intragastrically. Chow diet was administered at dark onset (7 p.m.) and chow intake was measured 3, 12 and 20 h later.

Determination of the effects of an acute dose of intragastric treatments in portal vein enterohormone secretion

Intragastric treatments were performed in two sets of animals. The first set comprised 10 female Wistar rats that received a specific intragastric dose of 1,10-Phenanthroline. The second set comprised 10 female Wistar rats that received an intragastric dose of (-)-Epicathechin. The same procedure (described earlier) was applied to both sets of rats. The animals were randomly divided into a control group and a treated group. The rats were fasted from 10 p.m. to 7 a.m. before treatment and anaesthetized 5 min later with either inhaled isoflurane (5% for induction, followed by 3% for maintenance) for the 1,10-Phenanthroline assay or pentobarbital (70 mg/kg) for the (-)-epicatechin assay. The abdominal cavity was incised through the midline and the portal vein was catheterized with a PE tube ( Inner Diameter(I.D.) 0.28 mm, Outer Diameter (O.D.) 0.61 mm; Becton Dickinson, Sparks, MD, USA) following a standard procedure. The catheter was fixed with cyanoacrylate and the abdominal cavity was closed with surgical clamps. The body temperature was kept constant at 37 °C by a heated surgical table and overhead lamps. At time zero, 200 µL of blood were obtained and the catheter was refilled with saline. The specific treatment or tap water as the vehicle was punctured into the forestomach. Two portal blood samples (200 μL) were taken after treatment (described in the results) and each time the catheter was refilled with heparinized 0.9% NaCl. The blood was transferred to heparinized tubes containing a 1:100 volume of a 1:1 mix of commercial Dipeptidyl peptidase-4 inhibitor (DPPIV, Millipore, Madrid, Spain), to which a serine protease inhibitor (cOmplete™ ULTRA Tablets, Roche, Barcelona, Spain) was added. Plasma was collected by centrifugation at 1500× *g* over 15 min at 4 °C and frozen immediately at −80 °C for enterohormone quantification. The rats were sacrificed by bilateral thoracotomy.

### 2.5. Enterohormone Quantification

We measured enterohormone secretions from intestinal segments and plasma with commercial kits. Total and active GLP-1 were measured with ELISA kits from Millipore (Cat No: EZGLPT1-36k and EGLP-35K, respectively, Burlington, MA, USA). PYY was measured using a fluorescent immunoassay kit (Cat No: FEK-059-03, Phoenix Pharmaceuticals, Burlingame, CA, USA). Total CCK was measured with an ELISA kit (Cat No: EKE-069-04, Phoenix Pharmaceuticals, Burlingame, CA, USA).

### 2.6. Statistical Analysis

Our results are presented as mean ± SEM. Data were analyzed with XLSTAT 2020.1 (Addinsoft, Barcelona, Spain) statistical software. Statistical differences were assessed by Student t-tests, and *p* < 0.05 was considered statistically significant.

## 3. Results

### 3.1. Stimulation with Specific Agonists of hTA2R5 Increases GLP1 and CCK Secretions, While Stimulation with Specific Agonists of hTA2R39 Tends to Increase PYY and Decrease CCK

**Figure 1:**
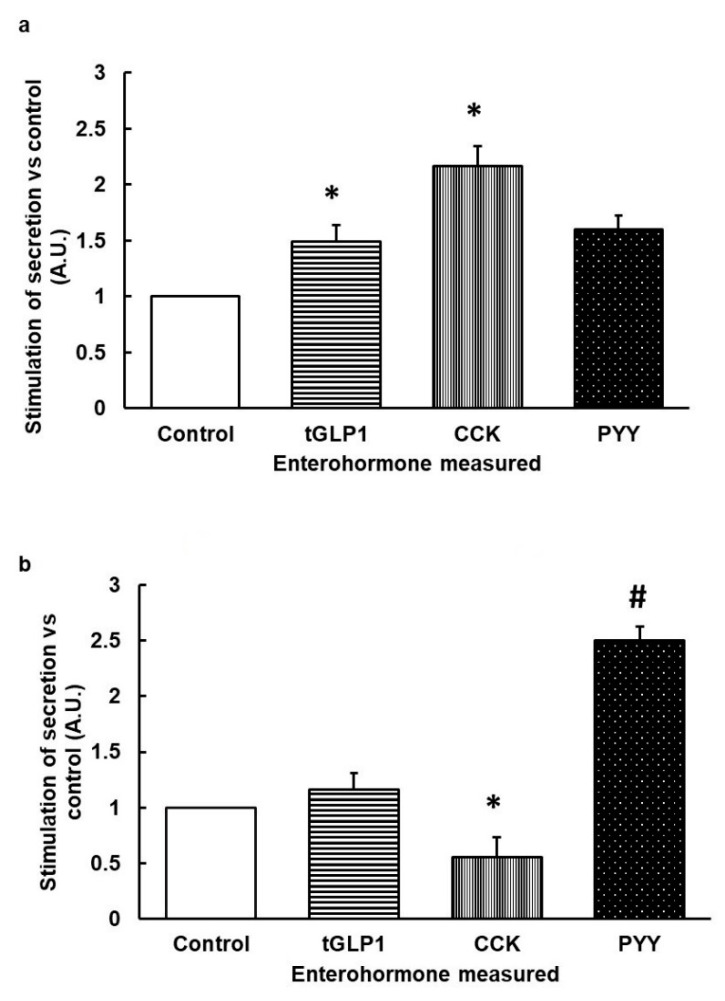
Ex vivo stimulation of enterohormone secretions induced by 1,10-Phenantroline (**a**) or Thiamine (**b**). Rat segments of duodenum (for cholecystokinin (CCK)) and ileum (for total glucagon-like peptide 1 (tGLP1) and peptide YY (PYY)) were treated with 1,10-Phenantroline 150 µM for 30 min (**a**) or Thiamine 1 mM for 30 min (**b**). Afterwards, medium was collected and respective enterohormones were quantified by ELISA (*n* = 6–10 segments). Results are calculated versus basal respective secretion in each hormone (Arbitrary Units, A.U.). Mean ± SEM. * denotes *p* < 0.05 vs. control; ^#^ indicate *p* < 0.1 vs. control.

We tested the stimulation of different receptors with different degrees of selectivity. In humans, the most selective bitter taste receptors are hTAS2R3, hTAS2R5, hTAS2R13 and hTAS2R8. 1,10-Phenantroline is the only selective agonist for the hTAS2R5 receptor [12]. That is, this compound does not bind to other receptors in human TAS2Rs. We assayed the extent to which 1,10-Phenantroline, at around its minimum effective dose for hTAS2R5 (defined in Table 1), stimulated explants of various segments of rat intestine. Figure 1a shows that 1,10-Phenantroline increased total GLP1 (tGLP1) and CCK secretion with no statistical differences on PYY secretions. 

According to Di Pizio et al. [12], hTAS2R39 is a less selective receptor. One of the selective agonists for it is Thiamine, though this compound also binds (h)TAS2R1 [11]. Treating rat intestine segments with the minimum effective dose of Thiamine for hTAS2R39 (Table 1) significantly reduced CCK secretion and tended to increase PYY secretion without affecting tGLP1 (Figure 1b).

### 3.2. When Bitter TAS2Rs Are Subjected to Simultaneous Stimulation, the Effect on Secretome Is Similar to the Effect on the Receptor with Lower EC50 Only

**Figure 2:**
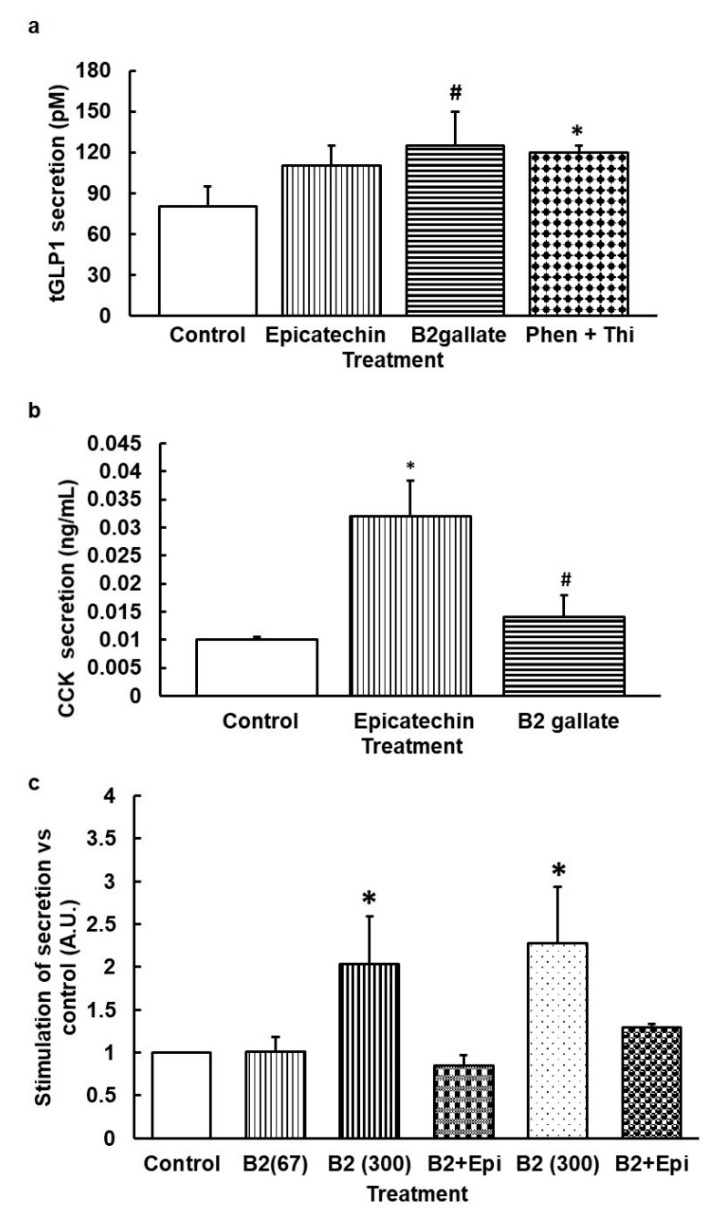
Ex vivo intestinal rat secretions induced by simultaneous stimulation by hTAS2R5 and hTAS2R39 agonist (**a**) and (**b**) or by Procyanidin B2 (**c**). (**a**) Segments of ileum were treated with epicatechin 1 mM, B2gallate 20 µM for 30 min or 1,10-Phenantroline 150 µM + Thiamine 1 mM for 45 min. (**b**) Segments of duodenum were treated with epicatechin 1 mM or B2gallate 20 μM for 30 min. (**c**) Segments of ileum (for tGLP1, vertical and squared lines) and duodenum (for CCK, dotted columns) were treated with B2 67 or 300 µM for 30 min, or B2 300 μM + epicatechin 1 mM for 45 min. Afterwards, medium was collected and respective enterohormones in the medium were quantified by ELISA (*n* = 6–10 segments). Mean ± SEM. (**c**) Results are calculated versus basal respective secretion in each hormone (Arbitrary Units, A.U.) * denotes *p* < 0.05 vs. control, ^#^ indicate *p* < 0.1 vs. control.

To understand responses closer to an in vivo situation, we assayed the simultaneous stimulation of hTAS2R5 and hTAS2R39 agonists with 1,10-Phenanthroline plus Thiamine in rat intestinal segments at doses close to their minimally effective concentration (Table 1). Figure 2a shows that ileal tGLP1 secretion was clearly stimulated by simultaneous stimulation with 1,10-Phenanthroline plus Thiamine. We then treated ileum segments with compounds that can bind both receptors but with a lower EC50 for hTAS2R5 than for hTAS2R39, i.e., B2gallate and (-)-epicatechin [19] (Table 1). B2 gallate tended to stimulate tGLP1 in ileum and CCK in the duodenum, while epicatechin significantly increased CCK secretion (Figure 2b).

A different response was obtained with EGCG, which binds with lower EC50 to hTAS2R39 and also binds to TAS2R5 (Table 1) and hTAS2R43 [19]. EGCG 30 µM limits tGLP1 secretion in ileum segments (79.41 ±7.90 pM vs. control: 81.14 ± 13.41 pM, *p* = 0.04).

Finally, we tested Procyanidin B2, which has not yet been defined as a ligand for any TAS2R (though it has been identified as bitter with a threshold recognition of 0.485 mM [21]). Figure 2c shows that Procyanidin B2 increases tGLP1 and CCK at a dose of 300 µM. Its combination with epicatechin prevents these effects (Figure 2c).

### 3.3. Stimulation with Agonists of hTAS2R14 Increases GLP1 Secretion

**Figure 3:**
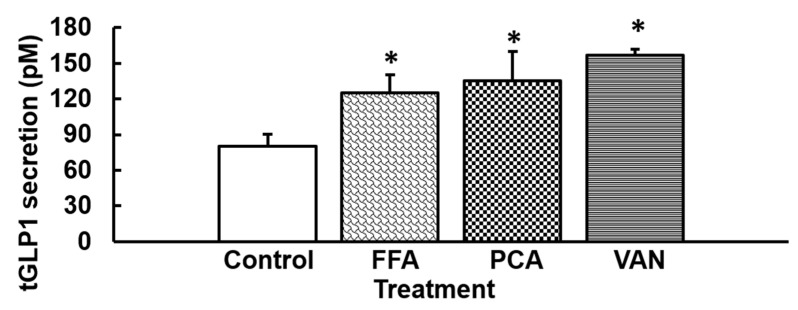
Ex vivo ileum rat total GLP1 secretion induced by hTAS2R14 agonists. Rat segments of ileum were treated with Flufenamic acid(FFA) 50 µM, protocatechuic acid(PCA) 300 µM or Vanillic acid(VAN) 300 µM for 30 min. Afterwards, the medium was collected and total GLP1 was quantified by ELISA (*n* = 6–10 segments). Mean ± SEM. * denotes *p* < 0.05 vs. control.

One of the most promiscuous bitter taste receptors in humans is TAS2R14 [12]. Flufenamic acid is a selective agonist for this receptor, with a minimally effective concentration of 10 µM [11]. Rat intestine segments treated with Flufenamic (FFA) acid increased tGLP1 secretion (Figure 3), reduced CCK (0.60 ± 0.17 vs. control (1.00 ± 0.26); *p* = 0.004) and did not significantly modify PYY (1.15 ± 0.47 vs. control (1.00 ± 0.35); *p* = 0.84).

Vanillic acid (VAN), another selective ligand for TAS2R14 with an EC50 of 151.17 µM [17] also increased tGLP1 secretion in ileum segments, as did protocatechuic acid (PCA), another ligand of TAS2R14 (and TAS2R30) [17] (Figure 3).

We also tested the effects of simultaneous activation with 1,10-Phenantroline (agonist for hTAS2R5) and Flufenamic acid (agonist for hTAS2R14), these being two stimulators of GLP1 secretion. Simultaneously treating ileum segments of rat intestine with the combination of Flufenamic acid (50 mM) and 1,10-Phenantroline (150 mM) did not induce tGLP1 secretion (control: 70.77 ± 7.47 vs. Phenantroline + Flufenamic acid: 104.01 ± 11.89 pM).

### 3.4. Agonists That Increase GLP1 and CCK Are More Effective in Limiting Food Intake

**Figure 4:**
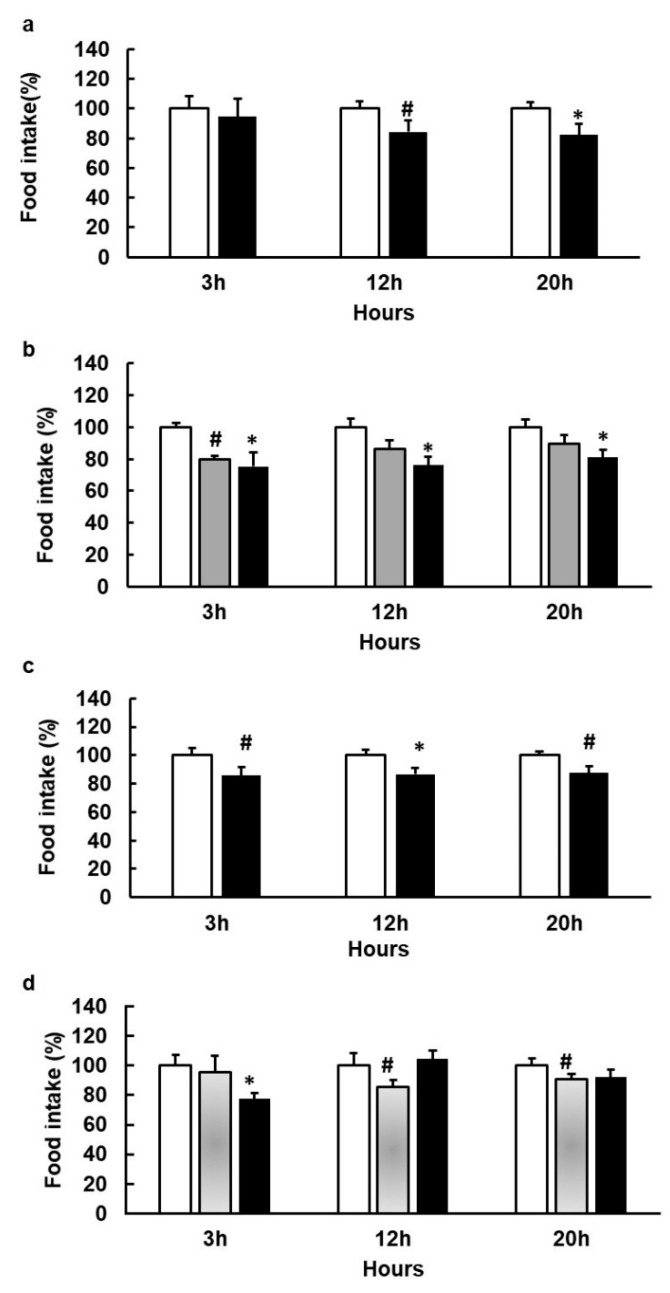
Food intake (FI) changes induced by acute doses of agonists of bitter taste receptors in female rats. Animals (*n* = 8–10/treatment) were treated one hour before dark period with an acute dose of 1,10-Phenantroline 200 mg/kg~290 µM (black columns) (**a**); 244 mg/kg~0.84 mM epicatechin (grey columns) or 300 mg/kg~1 mM epicatechin (black columns) (**b**); epicatechin + B2 + ECg (200 + 62 + 18 mg/kg) (black columns) (**c**); vanillic acid (252 mg/kg), grey columns) or Vanillic acid (252 mg/kg) + epicatechin (244 mg/kg) (black columns) (**d**); tap water as vehicle (white columns). Food intake was measured at the times indicated after the start of the dark period starts (Mean ± SEM). * denotes *p* < 0.05 vs. control; ^#^ indicate *p* < 0.1 vs. control.

Acute administration of 1,10-Phenantroline to rats led to a reduction in food intake (Figure 4a). The same treatment tended to induce higher levels of active GLP1 in the portal vein thirty minutes after administration (t0 = 1.00 ± 0.15; t30 = 1.63 ± 0.4, *p*-value = 0.09, arbitrary units relative to secretion at t0). There were no clear effects on PYY or CCK at this time point (CCK ng/mL: t0 = 0.42 ± 0.04; t30 = 0.7 ± 0.07 control; t30 = 0.63 ± 0.10 1,10-Phenantroline; PYY pg/mL: t30 = 40.1 ± 3.15 control; t30 = 47.7 ± 8.9 1,10-Phenantroline). Neither was there a clear effect on glucose (results not shown).

An acute dose of around 0.84 mM of epicatechin tended to inhibit food intake, whereas an acute dose of around 1 mM clearly limited food intake (Figure 4b). This dose, increased levels of active GLP1 in the portal vein forty minutes after treatment (t0 = 1.00 ± 0.19; t20 = 1.56 ± 0.5; t40 = 4.69 ± 1.8, *p*-value = 0.03, arbitrary units relative to secretion at t0) and CCK secretion (CCK ng/mL: t0 = 0.68 ± 0.02; t40 = 0.78 ± 0.13 control; t40 = 0.96 ± 0.12 epicatechin). However, it did not change glycemia (results not shown).

Procyanidin B2 at a dose of 0.11 mM did not affect food intake (Figure S1). However, the same dose of B2 plus epicatechin (0.74 mM) and epicatechin gallate (ECg) at a total dose of 0.84 mM did reduce food intake (Figure 4c).

Vanillic acid tended to reduce food intake (Figure 4d). The combination of epicatechin with vanillic acid, both of them at doses that reduce food intake, was only effective three hours after treatment and was not effective thereafter (Figure 4d).

### 3.5. Stronger Agonism of hTAS2R39 Than hTAS2R5 Can Stimulate Food Intake

Rats treated with Thiamine (a selective hTAS2R39 ligand) at a dose of 7.5 mM, which is much higher than the effective concentration (Table 1), did not modify food intake (Figure S2). Neither did epicatechin gallate, which also binds hTAS2R39, with an EC50 of 88.2 µM [22] (Figure S3).

**Figure 5:**
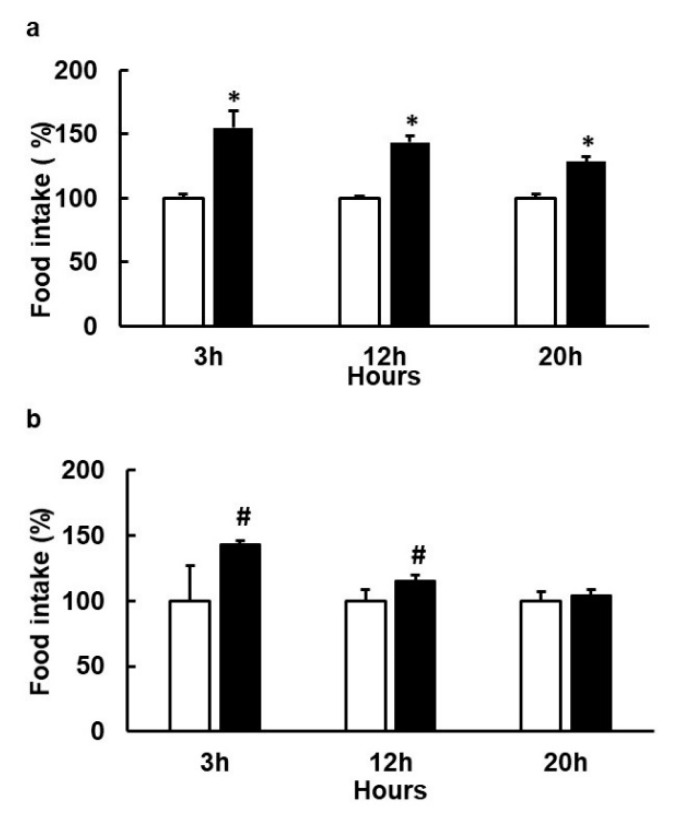
Changes in food intake (FI) induced by acute doses of agonists of bitter taste receptors in female rats. Animals (*n* = 8–10/treatment) were treated one hour before the dark period with an acute dose of epicatechin (234 mg/kg) + epicatechin gallate (14 mg/kg), a whole dose ~0.84 mM (black columns) (**a**); epicatechin (213 mg/kg) + B2 (62 mg/kg), a whole dose ~0.84 mM (**b**). White columns indicate the control group treated with tap water as vehicle. Food intake was measured at the times indicated after the start of the dark period starts (Mean ± SEM). * denotes *p* < 0.05 vs. control, ^#^ denotes *p* < 0.1 vs. control.

Stimulation with epicatechin 0.3 mM + 21.8 µM of EGCG (equivalent to simultaneous stimulation of hTAS2R39 and hTAS2R5) did not change food intake. We also found no effects when we doubled the epicatechin dose (0.78 mM) (Figure S4). Interestingly, when we added a selective hTAS2R39 agonist such as epicatechin gallate (at a dose that has no effect on food intake) to epicatechin to reach a total dose of 0.84 mM (at which epicatechin alone had no effect) we observed a stimulation of food intake (Figure 5a). This effect was also found with a simultaneous treatment of epicatechin plus procyanidin B2 (Figure 5b).

## 4. Discussion

We hypothesized that the specific stimulation of bitter taste receptors located in the gastrointestinal tract can produce a secretome that modulates food intake in rats. Knowledge of the role of these receptors in that location is scarce, though their ability to increase ghrelin secretion has been proven in two situations [15,16]. Here, we show that stimulation by some agonists for human TAS2R may be used as an on/off mechanism to elicit enterohormone secretions that modulate food intake in the organisms.

Our study is mainly based on the definitions by Meyerhof et al. [11] regarding the compounds that bind and activate human bitter taste receptors. To select the ligands, we worked with three receptors with different selectivities: a highly selective hTAS2R5, a moderately selective receptor such as hTAS2R39 and a highly promiscuous hTAS2R14. We stimulated intestinal segments with the agonists of the human TAS2Rs and measured their ability to induce gastrointestinal secretions that participate in the control of food intake. The most consolidated changes due to metabolic surgery (which modulates food intake) on the secretome in humans are increased GLP1 and PYY [4]. Here, we have found that in rats, the most effective changes in secretome for reducing food intake are increases in GLP1 and CCK, which do not affect PYY. The common denominator in these approaches is the increase in GLP1. In fact, the only approved drug for managing body weight via enterohormone mechanisms is based on GLP1 analogues such as liraglutide [23].

Increased GLP-1 is obtained with a specific agonist (1,10-Phenanthroline) or with agonists that preferentially bind to hTAS2R5 (epicatechin or B2 gallate). However, we also found an increase in GLP-1 via the stimulation of hTAS2R14—in this case together with a reduction in CCK, which, with vanillic acid, also tends to reduce food intake. There is no additive effect between epicatechin and vanillic acid and this cotreatment antagonizes their ability to stimulate tGLP1 secretion, which, as expected, limits their respective ability to inhibit food intake. Since we are working with theoretically defined receptors, we can postulate different interactions between these ligands to interfere with tGLP1 secretion. They could interfere intracellularly producing crosstalk between intracellular signaling [24], or there could be a desensitizing phenomenon, as has been defined for hTAS2R14 [25]. When the combination of epicatechin and vanillic acid was tested in vivo, at three hours we observed a reduction in food intake, probably due to epicatechin. Afterwards, when vanillic acids become effective (12 h onwards), the effects of the combination are lost, which could be due to heterologous desensitization. We obtained the same secretome profile (higher GLP1 and CCK) with procyanidin B2 at 300 µM. This procyanidin has not been shown to bind to any hTAS2R at the concentrations assayed (i.e., below 150 µM) [17]. When it was assayed at higher concentrations in intestinal segments, we found a similar secretome profile to that of epicatechin (a hTAS2R5 + hTAS2R39 agonist). Contrastingly, with the combination of procyanidin B2 with epicatechin assayed on intestinal segments, any differences on the secretion of neither GLP1 nor CCK were observed. This finding suggests that procyanidin B2 may act as a partial agonist of the effects of hTAS2R5. The effects of procyanidin B2 alone seem to correspond to those of agonists of hTAS2R5 and hTAS2R39 but in combination with another agonist, it cancels these effects [26]. When we used B2 alone with rats, we observed no effect. This was because we were working with a dose of 110 µM, which is closer to the dose of 67 µM (which was shown to be ineffective in the studies on secretome) than to the effective dose of 300 µM.

Stimulating only PYY secretion, as produced by specific hTAS2R39 stimulation, appears to be ineffective in reducing food intake in the rat. On the other hand, some combinations that preferentially target hTAS2R39 signaling (such as epicatechin plus epicatechin gallate) at a dose of over 0.84 mM do stimulate food intake. Previous studies with epicatechin showed a trend towards the stimulation of octanoyl ghrelin secretion in murine cells, while epicatechin gallate, a specific ligand for hTAS2R39, clearly stimulated octanoyl ghrelin—an effect that was abrogated by a specific antagonist for hTAS2R39 [15]. From these in vitro studies, it could be suggested that hTAS2R39 stimulates PYY and octanoyl ghrelin secretions. It was not possible to measure octanoyl ghrelin in our study because its presence in rat intestine is too low to be accurately measured. However, we cannot rule out the stimulation of ghrelin secretion in vivo that contributes to the orexigenic effect of this combination. We observed a similar stimulation of food intake when epicatechin plus procyanidin B2 was administered. We also treated ghrelin-producing cells with B2 or B2 gallate but octanoyl ghrelin secretion remained unchanged [15]. Therefore, if our hypothesis is that hTAS2R39 stimulation is related to the stimulation of food intake, we may suggest that B2 counteracts the effect of epicatechin in hTAS2R5 and allows only the stimulation of epicatechin in hTAS2R39.

Surprisingly, the combination of epicatechin plus procyanidin B2 and epicatechin gallate (all at non-effective doses for inhibiting food intake) leads to a reduction in food intake in the rat. To explain this finding, we postulate that all the ligands of hTAS2R39 in the mix compete amongst themselves at the level of the receptor or at other stages between the initial stimulation and the final effect on food intake, while the effects linked to hTAS2R5 remain unaltered. Nevertheless, we are unable to prove this with our data.

In addition to the different number of bitter taste receptors between species (25 in humans and 35 in rodents), there are different agonisms for the different sets of TAS2Rs possessed by each species [13]. 1,10-Phenantroline binds five bitter taste receptors in mice (mTas2r). Neither Thiamine nor Flufenamic acid has been tested in mice. Epicatechin binds two mTas2r while EGCG binds only one (mTas2144). In this study, we have been working on rats, about whom there is little information regarding TAS2R and their orthologues in humans, or their respective specificities against different ligands. Their proximity to mice can be used as a reference but extreme caution must be taken when extrapolating these results to humans. As an example, Avau et al. proved that intra-gastric stimulation induced a TAS2R-dependent delay in gastric emptying in mice that, when assayed in human volunteers, increased satiation [27]. Another aspect to address in the future should be gender effects since recently gender differences have been reported in humans [28,29]. Beyond these considerations, the importance of our study is the evidence that the stimulation with specific bitters produces enterohormone secretions linked to food intake modulation. However, specific attention must be paid to the possible differences between rat and human isoforms.

Finally, we used our hypothesis to explain the satiating effect of some doses of grape-seed derived procyanidins (GSPE), which we did not obtain when we used a very similar but slightly different (cocoa) extract [30]. Epicatechin, procyanidin B2, epicatechin gallate, vanillic acid and other ligands of hTAS2R5, hTAS2R14 and hTAS2R39 [17] are constituents of grape seeds. We showed that a grape-seed proanthocyanidin extract can increase GLP1 secretion, GIP and PYY [31,32] and limit food intake at doses above 350 mg/kg Bw [33]. Table S2 summarizes the abundance of ligands for hTAS2R5, hTAS2R14 and hTAS2R39 in GSPE, Cocoanox and the satiating combination (epicatechin + B2 + ECg). GSPE contains selective ligands for hTAS2R5 and very few amounts of ligands for hTAS2R14. The highest amounts of ligands are for hTAS2R5 and hTA2r39, together with selective ligands for hTAS2R39, which suggests competition by hTAS2R39 effects and enables the stimulation of enterohormones induced by hTAS2R5, which produces satiety. The case of Cocoanox resembles stimulation by epicatechin plus B2: either there is no effect or, depending on the ratio between both, food intake increases [30].

## 5. Conclusions

Food intake can be adjusted by gastrointestinal stimulation with compounds that bind to specific bitter taste receptors. This mechanism produces enterohormone secretions that can explain these effects on food intake. Specifically, the ligands of hTAS2R5 stimulation produce an anorexigenic effects in rats, whereas ligands of hTAS2R39 acts as an orexigenic. Further studies in humans are required to prove this strategy as means of controlling food intake.

## 6. Patents

There is a patent submitted on this manuscript P202030846.

## Figures and Tables

**Table 1:** Comparison of the doses of the agonists and ligands assayed in the study and the individual binding parameters defined for each.

Compound (hTAS2R)	EC_50_^1^ (μM)	Effective Concentration ^2^ (μM)	Dose Administered to Rats	Dose for Treatment of Intestine Explants
1,10-Phenantroline (hTAS2R5) [18]	Not defined	100	290 μM	150 μM
Thiamine(hTAS2R39) [11]	Not defined	1000	7.5 mM	1 mM
ECg (hTAS2R39) [19]	88.2	Not defined	31 μM	-
Epicatechin (hTAS2R5) [19]	3210	1000	0.84/1 mM	1 mM
Epicatechin (hTAS2R39) [19]	3800	-	-	-
B2 gallate(hTAS2R5) [17]	6.3	Not defined	-	20 μM
B2 gallate(hTAS2R39) [17]	9.11	Not defined	-	-
Epigallocatechin Gallate(EGCG) (hTAS2R5) [17]	12.3	-	-	-
EGCG(hTAS2R39) [17]	8.5	Not defined	21/43 μM	300 μM
EGCG(hTAS2R39) [20]	181.6	10	-	-
Flufenamic acid (hTAS2R14) [11]	Not defined	10	50 μM	-
Protocatecuhic acid(hTAS2R14) [17]	156	Not defined		300 μM
Vanillic acid (hTAS2R14) [17]	151	Not defined	1.5 mM	300 μM
Procyanidin B2 [21]	Not defined	485 μM ^3^	0.11 mM	67/300 μM

^1^ EC_50_: half-maximum effective concentrations. ^2^ Effective concentration: minimal concentration that elicited response. ^3^ Sensorial umbral, not effective concentration.

## Supplementary Materials

The following are available online at https://www.mdpi.com/2072-6643/12/12/3784/s1, Figure S1: Food intake after an acute dose of procyanidin B2 in female rats, Figure S2: Food intake after an acute dose of Thiamine in female rats, Figure S3: Food intake after an acute dose of epicatechin gallate in female rats, Figure S4: Food intake after an acute dose of epicatechin + EGCG in female rats. Table S1: Compounds tested for secretion in intestinal segments and food intake studies and their concentration, Table S2: Molarity of respective ligands of hTAS2R in GSPE and Cocoanox (mM).
