# Supplementary material for: Modulation of Food Intake by Differential TAS2R Stimulation in Rat
Source: Nutrients. 2020 Dec 10;12(12):3784. doi: 10.3390/nu12123784 (PMC7762996; doi:10.3390/nu12123784)
Supplement: Supplementary file 1 [file nutrients-12-03784-s001.pdf]

Supplementary material

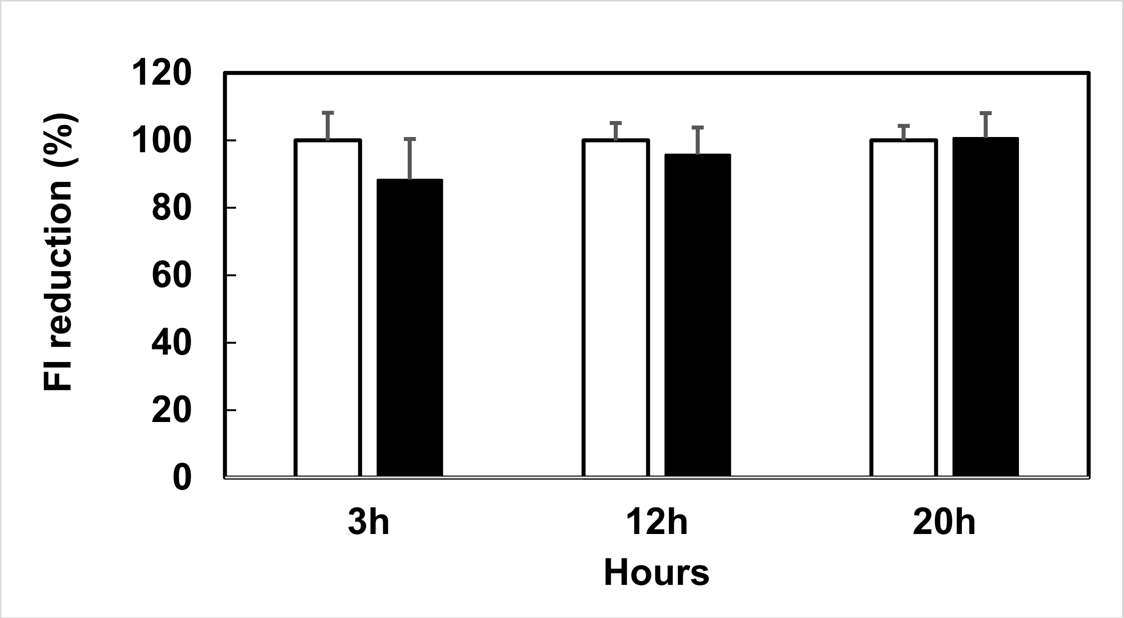

Figure s1. **Food intake after an acute dose of procyanidin B2 in female rats**. Animals were treated one hour before dark period with an acute dose of procyanidin B2, 62 mg/kg~ 0.11 mM (black columns) or tap water as vehicle (white columns). Food intake was measured at the times indicated since dark period starts.

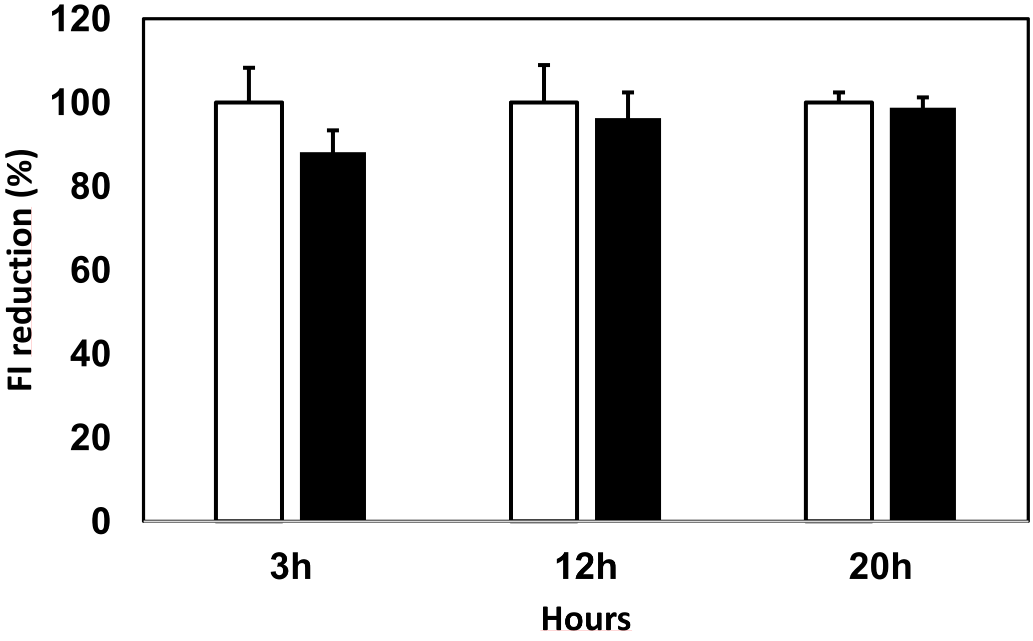

Figure s2. **Food intake after an acute dose of Thiamine in female rats**. Animals were treated one hour before dark period with an acute dose of Thiamine 2 g/kg~ 7.5 mM (black columns) or tap water as vehicle (white columns). Food intake was measured at the times indicated since dark period starts.

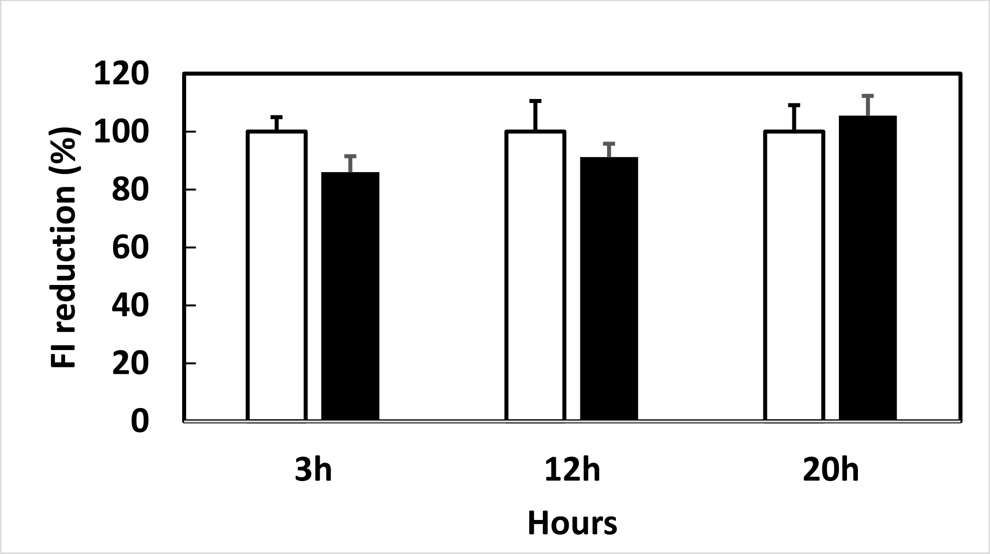

Figure s3. **Food intake after an acute dose of Epicatechin gallate in female rats**. Animals were treated one hour before dark period with an acute dose of Epicatechin gallate (14 mg/kg ~0.031 mM) (black columns) or tap water as vehicle (white columns). Food intake was measured at the times indicated since dark period starts.

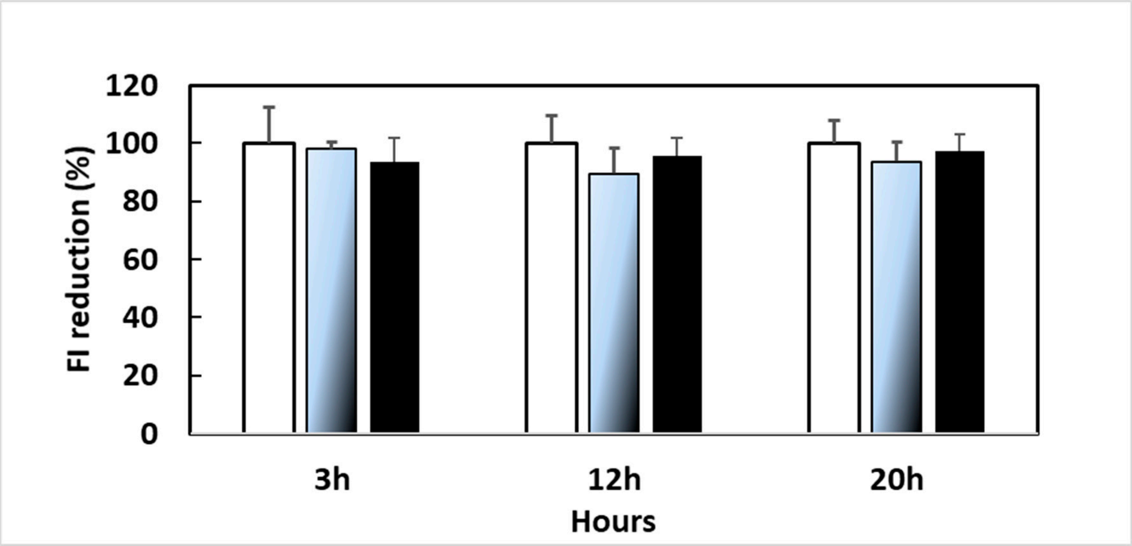

Figure S4. **Food intake after an acute dose of Epicatechin + EGCG in female rats**. Animals were treated one hour before dark period with an acute dose of Epicatechin 100 mg/kg + EGCG 10 mg/kg (grey columns). Or Epicatechin 200 mg/kg + EGCG 20 mg/kg

Table S1. Compounds tested for secretion in intestinal segments and food intake studies and their concentration.

Compound	Concentration
Tested in intestinal segments	
1,10-Phenanthroline	150 µM
Thiamine	1 mM
1,10-Phenanthroline + Thiamine	150 µM+1mM
Epicatechin	1 mM
Procyanidin B2 (B2)	67 or 300 µM
Epicatechin + B2	1 mM + 300 µM
Procyanidin B2 gallate	20 µM
Flufenamic acid	50 µM
Protocatechuic acid	300 µM
Vanillic acid	300 µM
Tested for food intake	
Epicatechin	244 mg/kg or 300 mg/kg
Procyanidin B2	62 mg/kg
Epicatechin + B2	213+62 mg/kg
Epicatechin + B2 + Epicatechin gallate	200+62+18 mg/kg
Vanillic acid	252 mg/kg
Epicatechin + Vanillic acid	252+244 mg/kg
Epicatechin + Epigallocatechin gallate	100+10 mg/kg or 200+20 mg/kg
Epicatechin + Epicatechin gallate	234+14 mg/kg

Table S2. Molarity of respective ligands of hTAS2R in GSPE and Cocoanox (mM).

	GSPE	Cocoanox	Epicatechin + B2 + Epicatechin gallate
Selective for hTAS2R5 (Trimer)	32.8	-	-
hTAS2R5 and 39 (Epicatechin, B2g, EgCg)	244.0	66.7	0.69
Selective for hTAS2R39 (ECg)	125.0	-	0.04
Selective for hTAS2R14 (Vanillic, PCA)	7.1	-	-
B2 (dimeric procyanidin)	249.3	29.1	0.11
